# Decorating MnO_2_ nanosheets on MOF-derived Co_3_O_4_ as a battery-type electrode for hybrid supercapacitors†

**DOI:** 10.1039/d2ra05603h

**Published:** 2022-10-11

**Authors:** S. Kishore Babu, B. Gunasekaran, M. Sridharan, T. Vijayakumar

**Affiliations:** Department of Physics and Nanotechnology, College of Engineering and Technology, Faculty of Engineering and Technology, SRM Institute of Science and Technology SRM Nagar, Kattankulathur – 603 203, Kancheepuram Chennai Tamil Nadu India gunasekb@srmist.edu.in; Electrochemical Energy Laboratory, Department of Chemistry, College of Engineering and Technology, Faculty of Engineering and Technology, SRM Institute of Science and Technology Kattankulathur – 603 203, Kancheepuram Tamil Nadu India; Futuristic Materials Research Centre for Planetary Exploration, Department of Physics and Nanotechnology, College of Engineering and Technology, Faculty of Engineering and Technology, SRM Institute of Science and Technology Kattankulathur – 603 203, Kancheepuram Tamil Nadu India

## Abstract

Metal–organic framework-derived materials are now considered potential next-generation electrode materials for supercapacitors. In this present investigation, Co_3_O_4_@MnO_2_ nanosheets are synthesized using ZIF-67, which is used as a sacrificial template through a facile hydrothermal method. The unique vertically grown nanosheets provide an effective pathway for rapidly transporting electrons and ions. As a result, the ZIF-67 derived Co_3_O_4_@MnO_2_-3 electrode material shows a high specific capacitance of 768 C g^−1^ at 1 A g^−1^ current density with outstanding cycling stability (86% retention after 5000 cycles) and the porous structure of the material has a good BET surface area of 160.8 m^2^ g^−1^. As a hybrid supercapacitor, Co_3_O_4_@MnO_2_-3//activated carbon exhibits a high specific capacitance (82.9 C g^−1^) and long cycle life (85.5% retention after 5000 cycles). Moreover, a high energy density of 60.17 W h kg^−1^ and power density of 2674.37 W kg^−1^ has been achieved. This attractive performance reveals that Co_3_O_4_@MnO_2_ nanosheets could find potential applications as an electrode material for high-performance hybrid supercapacitors.

## Abstract 2

Metal–organic framework-derived materials are now considered potential next-generation electrode materials for supercapacitors.

## Graphical abstract

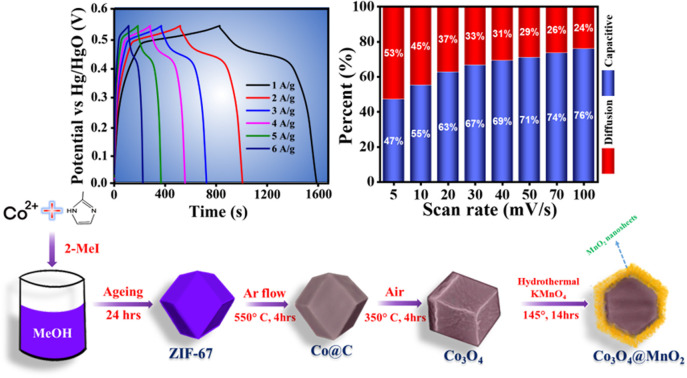

## 1. Introduction

Over the last few years, much emphasis has been placed on developing lightweight, versatile, and environmentally safe solid-state energy storage technologies in consumer electronics, slide displays, and miniature medical implants.^1–7^ The battery and the supercapacitor are the most effective sources of energy. However, the batteries' bulkiness, slow charge–discharge rate, and short life period restrict their use in wearable and portable devices.^8–10^ Supercapacitors have recently gained much attention, particularly in the automotive industry, because of their key characteristics like high power density, lightweight, fast charging–discharging rates, reliable handling, and long lifetime. Based on their charge storage mechanism, supercapacitors are grouped into two categories: electrical double-layer capacitors (EDLCs), which generally use carbon-active materials, and pseudocapacitors, which use redox-active materials.^11,12^ Due to their high energy density with rapidly reversible surface redox processes, pseudocapacitors have considerable potential as supercapacitor options in the future. On the other side, several pseudocapacitive materials support one of two inadequate cycling stability and poor conductivity.^13–17^ Due to their characteristics of considerable specific capacitance and high-rate capacitance, transition metal oxides (RuO_2_, Co_3_O_4_, SnO_2_, and MnO_2_, among others) have prompted widespread attention in the field of pseudocapacitive electrode materials with substantial specific capacitance.^18–20^ MnO_2_ is one of the most used materials for supercapacitors. It has a wide selection of high attributes such as low cost, environmental friendliness, abundant reserves, and a high theoretical potential capacity.^21,22^ However, the weak electrical conductivity of MnO_2_ and the practical, specific capacitance of the product is significantly lower than its theoretical result (1370 F g^−1^).^23^ The distinctive structural properties can be combined in such electrodes to improve rate and cycle capability.

Additionally, MnO_2_ materials have a limited loading of active materials, resulting in low energy density because of the low number of active sites. As a result, increasing the electrochemical utilization of MnO_2_'s pseudocapacitance by rationally constructing MnO_2_-based electrodes with innovative architectures and dependable electric connections remains a significant problem. Directly growing innovative integrated array mechanisms are fascinating in conducting substances for supercapacitors. It will provide synergistic effects from their respective materials, achieving high power density, energy density, and long cycle life.^24^ Co_3_O_4_ has become the subject of extensive research and development due to its low cost and high theoretical capacitance. Aside from that, introducing beneficial ions into Co_3_O_4_ and its composites can improve electrochemical performance due to the increased electric conductivity and the enhancement of faradaic redox reactions that result from this process. However, single-phase Co_3_O_4_ cannot meet the demands of actual applications due to its low capacity and inferior cycling stability, which are caused by few electroactive sites, weak ion diffusion, and limited electric conductivity.^25,26^ An alternative solution is a metal–organic framework (MOF) based synthesis of Co_3_O_4_ to satisfy these difficulties. It is possible to enhance the performance of the material by using the MOF's porous structure.^27–31^

This study aimed to investigate the electrochemical performance of MnO_2_ nanosheets decorated on MOF-derived Co_3_O_4_ synthesized in a facile two-step procedure. To construct Co_3_O_4_@MnO_2_ nanosheets, a sacrificial template (ZIF-67) was used to prepare the Co_3_O_4_. Then MnO_2_ nanosheet arrays were anchored to its surface using a hydrothermal technique. This unique structural design makes it possible to store a lot of energy and improve electrochemical performance by increasing the surface area.

## 2. Experimental

### 2.1. Preparation of Co_3_O_4_

The ZIF-67 precursor was used as a sacrificial template in a two-step process to synthesize Co_3_O_4_. The first step involves the synthesis of the precursor ZIF-67 using a standard preparation method. Where 1.74 g of cobalt nitrate and 1.968 g of 2-methylimidazole have dissolved in 60 mL and 20 mL of methanol, respectively. The two solutions were then combined and gently shaken for 7 minutes. The mixed solution was then stored at room temperature for 48 hours. The resulting precipitate was separated using a centrifuge, rinsed with methanol and dried for 12 hours at 80 °C to obtain the precursor ZIF-67. The second step entails the utilization of the synthesized ZIF-67 as a sacrificial template. Then the sample was calcinated in an argon atmosphere for 4 hours with a heating rate of 1 °C min^−1^ up to 550 °C and in an air atmosphere for 4 hours up to 350 °C. The final collected dark powder was named ZIF-67 derived Co_3_O_4_.

### 2.2 Preparation of Co_3_O_4_@MnO_2_

To prepare Co_3_O_4_@MnO_2_, 45 mg of prepared Co_3_O_4_ powder were ultrasonically dispersed in 30 mL of DI water. Then the prepared solutions were mixed with 30 mL DI water containing 30 mg, 45 mg, and 60 mg of potassium permanganate, respectively and placed into the stainless-steel Teflon reaction kettle to react for 14 hours at 145 °C. The final grey powders collected by centrifugation and drying at overnight are named as Co_3_O_4_@MnO_2_-1, Co_3_O_4_@MnO_2_-2 and Co_3_O_4_@MnO_2_-3 corresponding to 30 mg, 45 mg, and 60 mg of KMnO_4_ respectively.

### 2.3 Material characterizations and electrochemical measurements

The crystallographic structure and surface element compositions of prepared samples were characterized by XRD (BRUKER USA D8 Advance, Davinci) and X-ray photoelectron spectroscopy (PHI Versaprobe III). A field-emission scanning electron microscope (Thermosceintific Apreo S) and high-resolution transmission electron microscope (JEOL Japan, JEM-2100 Plus) were used to examine the microstructure and morphology of the synthesized materials. A thermogravimetric analyzer determines the thermal stability of the sample. Thermogravimetric measurements are taken in a nitrogen atmosphere from 50 to 800 degrees Celsius at a linear heating rate of 10 degrees Celsius per minute. Furthermore, FTIR was used to characterize the various bonds present on the surfaces of the prepared material (SHIMADZU, IRTRACER 100). The electrochemical performances of all prepared electrodes were performed in 1 M KOH using an electrochemical workstation (Biologic-SP200 Potentiostat). The three-electrode assessment used the active material as the working electrode, platinum as the counter electrode and Hg/HgO as the reference electrode. The two-electrode evaluation was carried out with Co_3_O_4_@MnO_2_-3 as the positive electrode and activated carbon as the negative electrode. The electrode material for the assessment was prepared by evenly blending the active material, conductive substance (carbon black), and binder (NMP) in an 8 : 1 : 1 ratio and then coating it on half of the 0.5 × 1 cm nickel foam.

The following formula determines the specific capacitance (*C*_p_) from the chronopotentiometry charge–discharge curves.^32^1
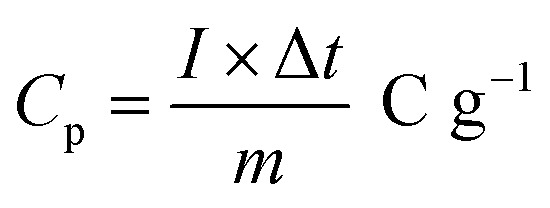
where *Im* is the current density (A g^−1^), Δ*t* is the discharge time (s), and Δ*V* is the voltage window (V).

The following equation is to determine energy density (*E*) and power density (*P*).^33^2
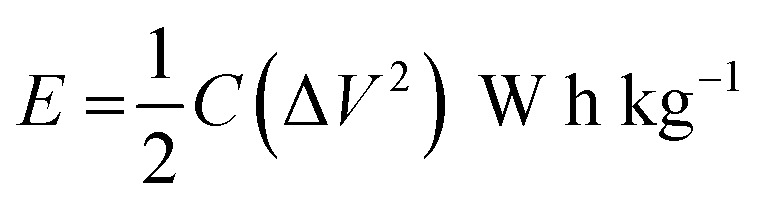
3
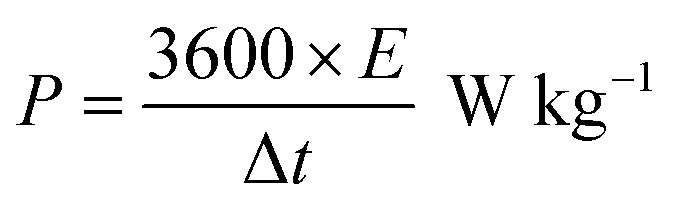
where *E*, *C*, Δ*V*, *P* and Δ*t* are the energy density, specific capacitance, potential window, power density and discharge time.

## 3. Results and discussions

### 3.1 Structural characteristics

**Fig. 1:**
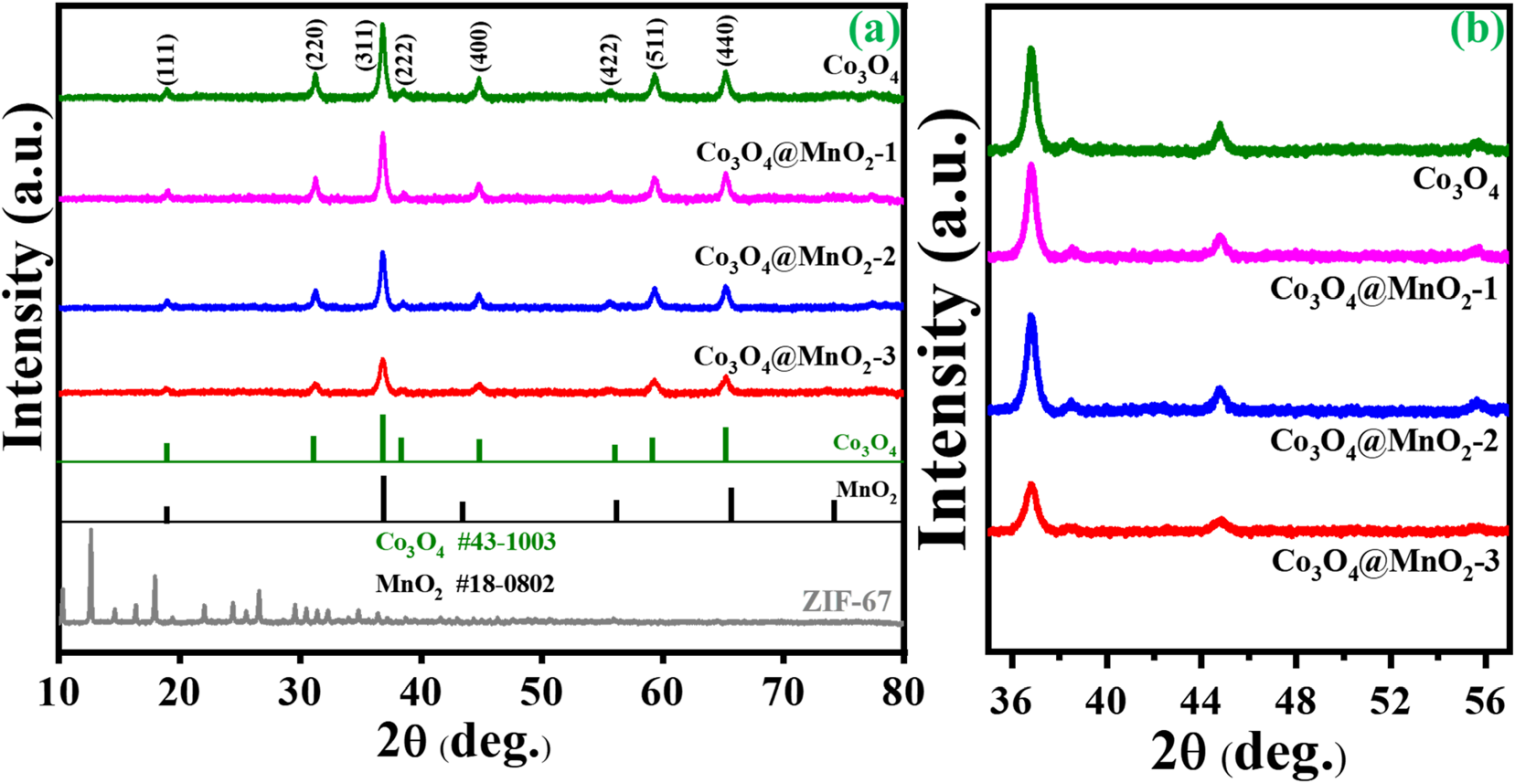
(a) Wide angle XRD of ZIF-67, Co_3_O_4_, Co_3_O_4_@MnO_2_-1, Co_3_O_4_@MnO_2_-2, Co_3_O_4_@MnO_2_-3 and (b) low angle XRD in the 2*θ* range of 36° to 56°.

**Fig. 2:**
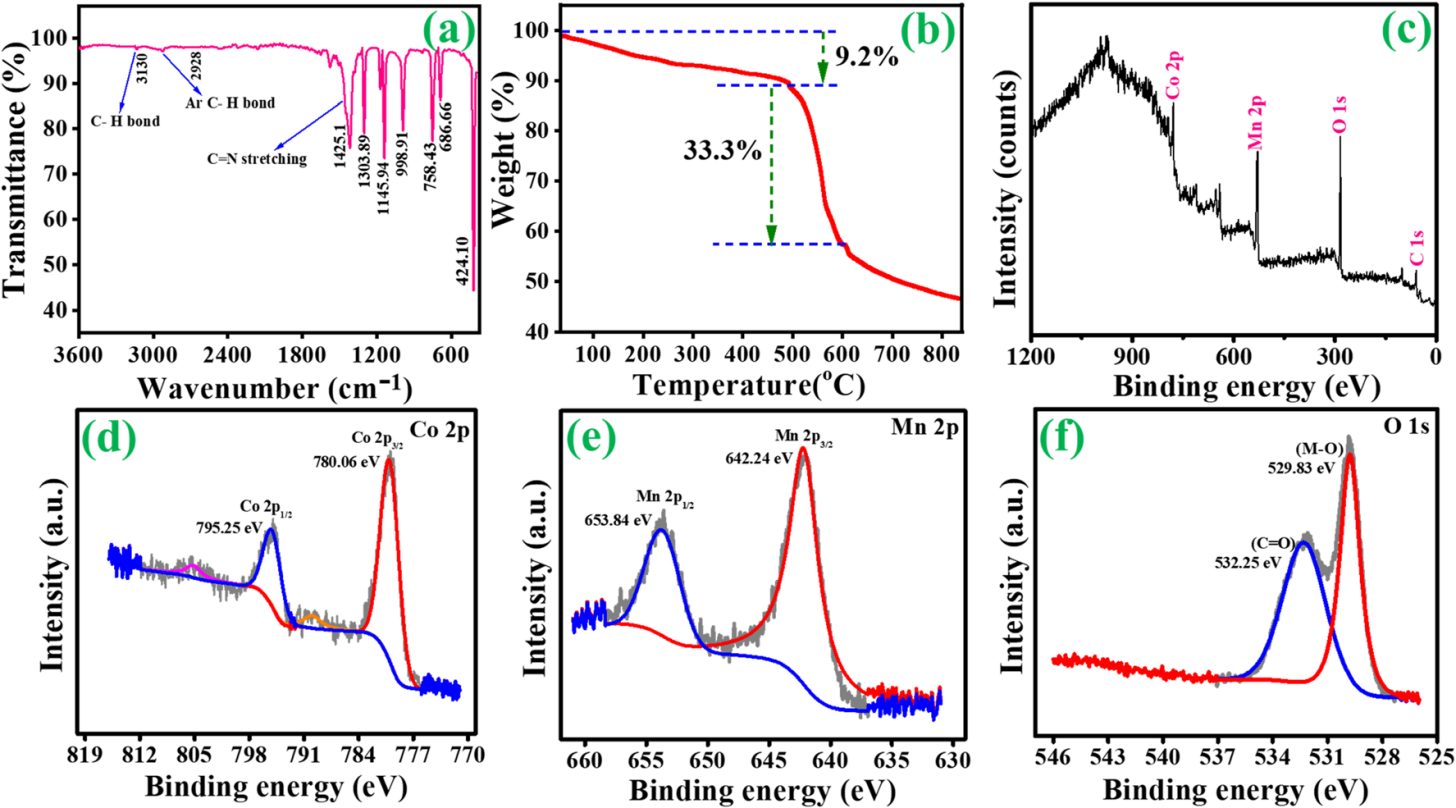
(a) FT-IR analysis of ZIF-67, (b) TGA analysis of ZIF-67 and (c–f) X-ray photoelectron spectra of Co_3_O_4_@MnO_2_-3.

An X-ray diffraction spectrometer (XRD) was used to examine the as-prepared samples' crystal structure and phase purity. Fig. 1(a) depicts the findings of ZIF-67. Certain significant diffraction peaks occurred in 2*θ* = 10.5°, 12.7°, 14.6°, 16.6°, 18.2° and 26.1 as seen in the resulting pattern, which may be independently confirmed with the sample ZIF-67 and are consistent with the literature.^34^ Fig. S1†(a) shows the XRD peaks of the intermediate Co/C product, which is consistent with earlier studies.^35^ XRD patterns of MOF-derived Co_3_O_4_ and Co_3_O_4_/MnO_2_-1, Co_3_O_4_/MnO_2_-2 and Co_3_O_4_/MnO_2_-3 are shown in Fig. 1(a and b). The characteristic peaks at 2*θ* = 19°, 31.2°, 36.8°, 38.5°, 44.8°, 55.6°, 59.3° and 65.2° can be assigned to the (111), (220), (311), (222), (400), (422), (511) and (440) lattice plane of cubic cobalt oxide phases (JCPDS no. 43-1003).^36^ Further, on decorating with MnO_2_ nanosheets, no notable characteristic peaks were found in the composition of Co_3_O_4_@MnO_2_ but with decreasing intensity. This decrease is attributed to the amorphous nature of MnO_2_ layers (JCPDS no. 18-0802) present over the surface, increasing the conc. of KMnO_4_.^37–39^ The low angle XRD pattern clearly shows the intensity decreased with increasing the concentration of KMnO_4_ shown in Fig. 1(b). FT-IR analysis was performed to identify the functional group in the prepared samples. In Fig. 2(a), ZIF-67 shows that peaks from 400 to 1400 cm^−1^ are associated with imidazolate moieties' stretching and bending vibrations.^40^ A minor peak at 1425.1 cm^−1^ was due to the stretching mode of the C=N bond in 2 methylimidazoles. Moreover, two minor peaks, 3130 and 2928 cm^−1^ were attributed to the stretching of C–H from the aliphatic methyl group and aromatic ring of 2 methylimidazoles, respectively. Also, FT-IR analysis for MOF-derived Co_3_O_4_, Co_3_O_4_@MnO_2_-1, Co_3_O_4_@MnO_2_-2 and Co_3_O_4_@MnO_2_-3 are shown in Fig S2.† The band at 3410 cm^−1^ indicates the O–H stretching of water, whereas the weak band at 1647 cm^−1^ is associated with the O–H group of bending vibration in the molecule of absorbed water.^41^ And the bands approximately at 1417 and 1090 cm^−1^ match the coordination of Co by the O–H. However, the prominent peaks at 592 cm^−1^ and 520 cm^−1^ are attributed to the stretching vibrations of M–O or M–O–M (M = Co, Mn).^42^

Fig. 2(b) shows the TGA analysis of ZIF-67. For ZIF-67, three decomposition steps occur under the N_2_ atmosphere TGA analysis. The absorption of methanol molecules observed on the surface of ZIF-67 at temperatures below 150 °C is the first stage of weight loss. The carbonization of 2-methylimidazole molecules in ZIF-67 pores from 250 to 490 °C causes the second stage (9.2%). The third stage's significant loss (33.3%) occurs when the temperature reaches a specific point. At this point, the organic groups and ZIF-67 dodecahedrons break down, revealing the final phase above 500 °C. The resulting calcined materials' oxidation states and chemical compositions were confirmed using XPS. The XPS survey spectra of Co_3_O_4_@MnO_2_-3 are shown in Fig. 2(c). As shown in Fig. 2(d), the high-resolution XPS spectra of Co 2p can be fitted into two primary peaks at 780.06 and 795.25 eV and can be associated with the binding energies of Co 2p_3/2_ and Co 2p_1/2_, respectively. The lower two peaks, 789 and 805.35 eV, can be assigned to the binding energies of 2p_3/2_ and 2p_1/2_ of Co(ii) and Co(iii). These results reveal the presence of the Co_3_O_4_ phase in the prepared sample.^43,44^ The high-resolution XPS spectra obtained from Mn 2p are shown in Fig. 2(e). The primary two peaks are centred at 642.24 and 653.84 eV, respectively, with a spin energy separation of 11.6 eV corresponding to Mn(iv). These findings are based on the electronic orbits of Mn 2p_3/2_ and 2p_1/2_, indicating that the compounds are in the Mn(iv) state.^45^ In Fig. 2(f), the binding energy peak at 532.2 eV is attributed to the oxygen atoms in the hydroxyl groups. In contrast, the intense peak at 529.8 belongs to the oxygen atoms in the Co_3_O_4_@MnO_2_-3 chemical compositions.^46,47^

### 3.2 Morphological analysis

**Fig. 3:**
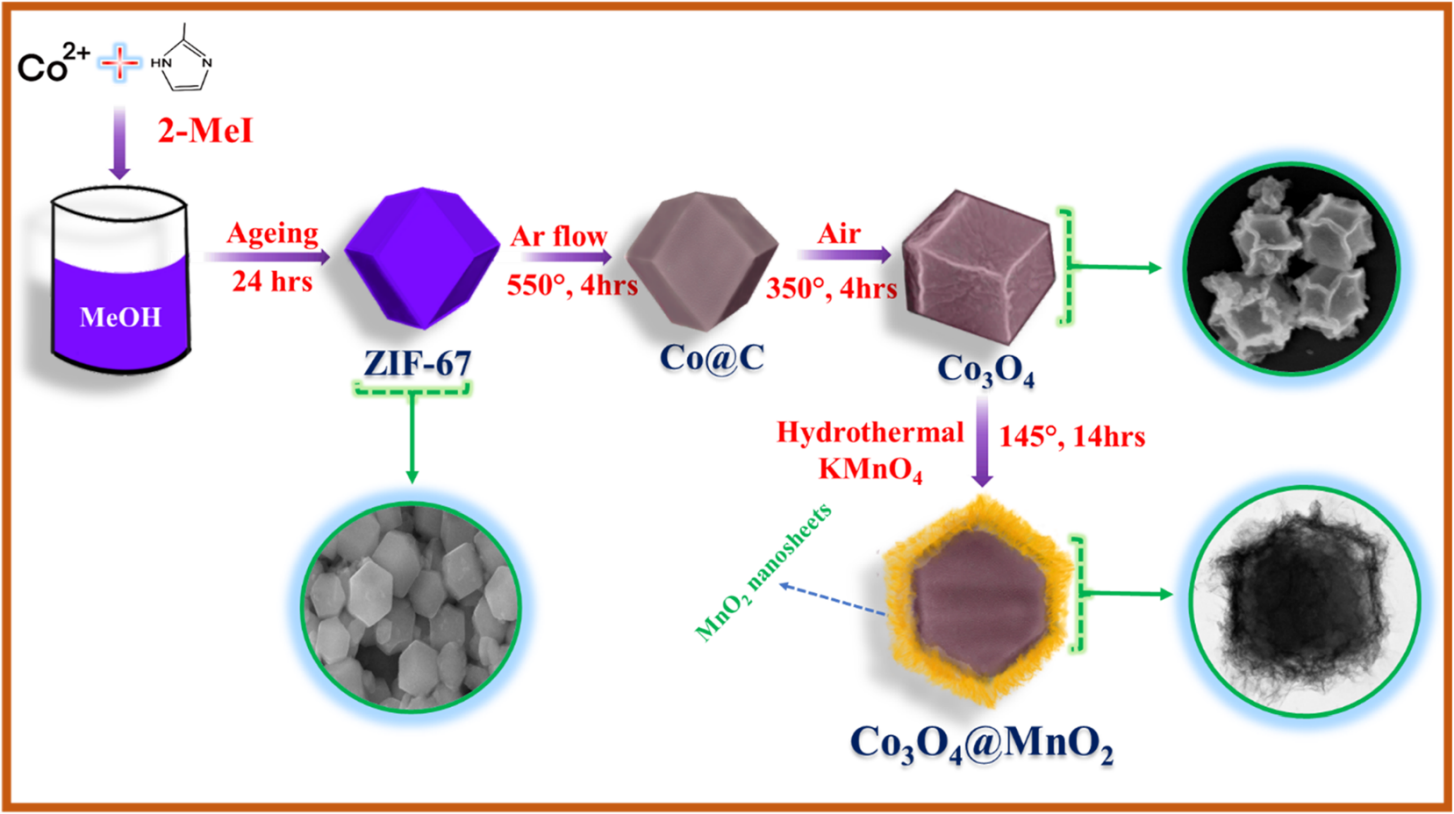
Schematic diagram of the synthesis of Co_3_O_4_@MnO_2_ core–shell structure.

**Fig. 4:**
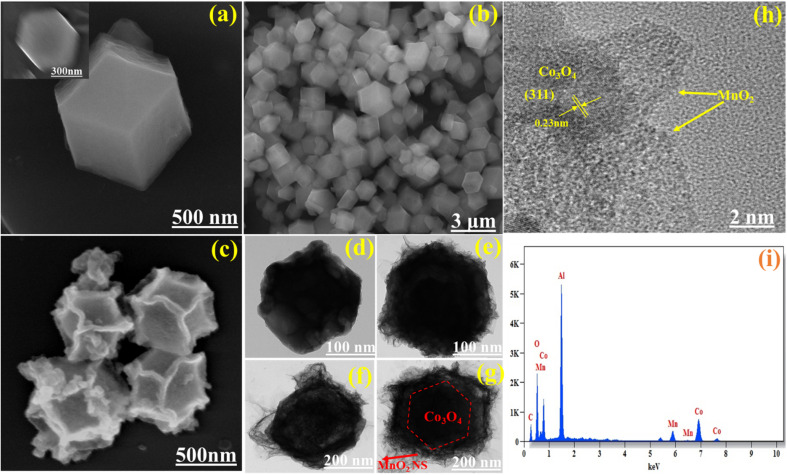
(a & b) SEM image of ZIF-67 with low magnification (inset), (c) Co_3_O_4_, TEM image of (d) Co_3_O_4_, (e) Co_3_O_4_@MnO_2_-1, (f) Co_3_O_4_@MnO_2_-2 and (g) Co_3_O_4_@MnO_2_-3, (h) HR-TEM image of Co_3_O_4_@MnO_2_-3, and (i) EDX pattern of Co_3_O_4_@MnO_2_-3.

As shown in the schematic illustration in Fig. 3, The dodecahedral ZIF-67 was used as a template for the synthesis of Co_3_O_4_ after pyrolysis at 550 °C in an argon atmosphere and calcinated at 350 °C in air. Then, Co_3_O_4_ was used as the substrate to grow MnO_2_ nanosheets *via* a hydrothermal process at 140 °C to form a Co_3_O_4_@MnO_2_. This formation is well proved by SEM and HR-TEM results. As illustrated in Fig. 4(a and b), the ZIF-67 has a standard form and a smooth surface. In Fig S1†(b), we show the TEM image of the Co/C sample and it is found that the sample is stable after calcination at 550 °C under an inert gas atmosphere. In contrast, the Co_3_O_4_ driven by ZIF-67 has a rough surface, illustrated in Fig. 4(c) and the TEM image is illustrated in Fig. 4(d) due to the collapse of a portion of the MOF frame during the calcination process. As shown in Fig. 4(e–g), the staggered MnO_2_ nanosheets clusters vertically grown on the surface of Co_3_O_4_ as the concentration of KMnO_4_ increases, forming a structure similar to dodecahedral. The TEM images reflect the unique hierarchical Co_3_O_4_@MnO_2_ nanostructure containing a core of Co_3_O_4_ dodecahedral and a shell of MnO_2_ nanosheets. The interface contacts between the black core Co_3_O_4_ and grey shell MnO_2_ nanosheet arrays were seen vertically. Surprisingly, the concentration of KMnO_4_ affects the distribution of MnO_2_ nanosheets on the Co_3_O_4_ surface during the hydrothermal process. As illustrated in Fig. 4(e), the surface of the Co_3_O_4_@MnO_2_-1 sample was only covered by partial and uneven MnO_2_ nanosheets due to insufficient KMnO_4_. However, excessive KMnO_4_ results in the overlapping of MnO_2_ nanosheets on a portion of the Co_3_O_4_ surface, as well as partially formed MnO_2_ nanosheets, as illustrated in Fig. 4(f) Co_3_O_4_@MnO_2_-2 sample.

In comparison, the composite Co_3_O_4_@MnO_2_-3, MnO_2_ nanosheet arrays with staggered and orderly vertical growth exhibit an appealing and satisfying morphology, as illustrated in Fig. 4(g), which corresponds to the superior electrochemical performance. Additionally, as illustrated in Fig. 4(h), the interplanar crystal spacing of the well-defined lattice fringes is 0.23 nm, which corresponds to the (3 1 1) plane of cubic Co_3_O_4_ and amorphous MnO_2_ overlayer on the surface of Co_3_O_4_ core could be clearly identified. Furthermore, the energy dispersive spectroscopy (EDS) indicates Co, Mn, O, and C presented in the Co_3_O_4_@MnO_2_-3 shown in Fig. 4(i), which is also consistent with the XPS results. In Fig. S3,† the element mapping images revealed a homogeneous distribution of all elements, confirming that the Co_3_O_4_@MnO_2_-3 was successfully synthesized.

### 3.3 Surface area analysis

**Fig. 5:**
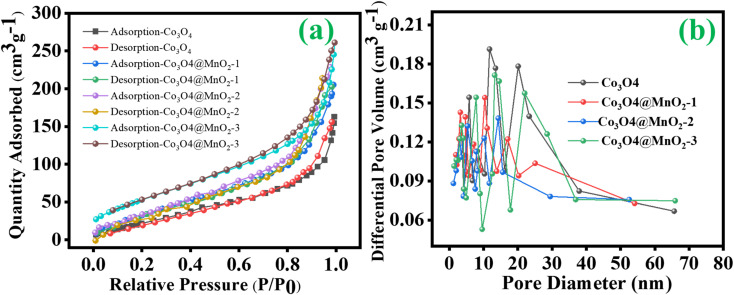
(a) BET analysis and (b) BJH pore size distribution curves of ZIF-67 derived Co_3_O_4_, Co_3_O_4_@MnO_2_-1, Co_3_O_4_@MnO_2_-2 and Co_3_O_4_@MnO_2_-3.

Furthermore, the specific surface area and porous characteristics of ZIF-67-derived Co_3_O_4_, Co_3_O_4_@MnO_2_-1, Co_3_O_4_@MnO_2_-2 and Co_3_O_4_@MnO_2_-3 were determined using N_2_ isothermal adsorption–desorption measurements. The prepared samples were typical Type-IV isotherms with an H3 hysteresis loop (Fig. 5(a)). According to the Brunauer–Emmett–Teller (BET) method, the determined specific surface area of Co_3_O_4_@MnO_2_-3 is 160.8 m^2^ g^−1^, while Co_3_O_4_@MnO_2_-1 (121.3 m^2^ g^−1^) and Co_3_O_4_@MnO_2_-2 (138.5 m^2^ g^−1^) and much better than ZIF-derived Co_3_O_4_ (109 m^2^ g^−1^), emphasizing the superiority of the design of the core–shell structure. It can be observed that the specific surface area and porosity of the prepared materials are greatly improved after decorating MnO_2_ nanosheets on the surface of cobalt oxide. Also, the mesopores structure can provide a plentiful ion transport/charge storage, which enhances the pseudocapacitance. The Barrett–Joyner–Halenda (BJH) technique determined the pore size distribution, as shown in Fig. 5(b) and reveals the mesoporous nature of all prepared samples. And the respective average pore size was obtained for Co_3_O_4_@MnO_2_-3 at around 8 nm, whereas ZIF-derived Co_3_O_4_, Co_3_O_4_@MnO_2_-1 and Co_3_O_4_@MnO_2_-2 show pore size of 10, 9.2 and 8.6 nm respectively. The large specific surface area provides abundant opportunities for the electrode and the electrolyte to make complete contact, which builds a strong foundation for the excellent electrochemical performance of Co_3_O_4_@MnO_2_, which can be attributed to the material.

### 3.4 Electrochemical performance

**Fig. 6:**
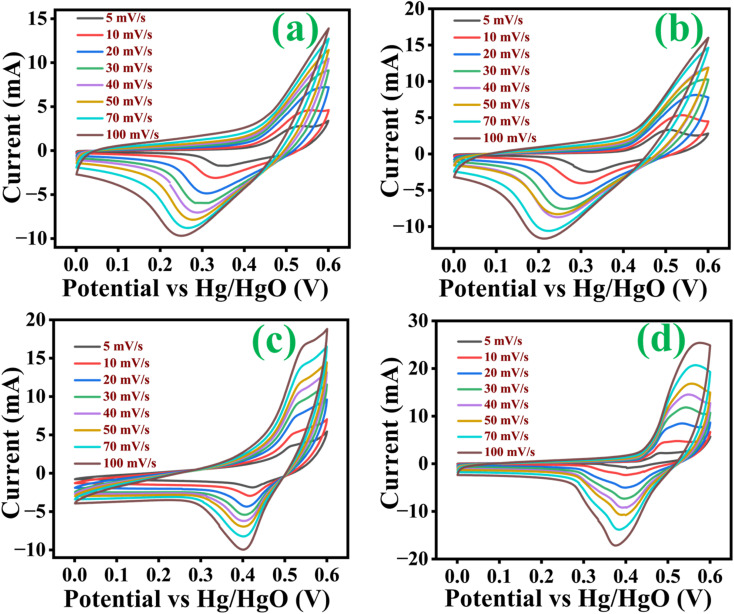
Cyclic voltammetry curves at several scan rate of (a) Co_3_O_4_, (b) Co_3_O_4_@MnO_2_-1, (c) Co_3_O_4_@MnO_2_-2 and (d) Co_3_O_4_@MnO_2_-3.

Cyclic voltammetry (CV) curves of all electrodes with a potential window of 0 V to 0.6 V at scan rates 5, 10, 20, 30, 40, 50, 70 and 100 mV s^−1^ were taken. Fig. 6(a–d) represents the CV curve of Co_3_O_4_, Co_3_O_4_@MnO_2_-1, Co_3_O_4_@MnO_2_-2 and Co_3_O_4_@MnO_2_-3. The two mild redox peaks are observed in the CV curve of Co_3_O_4_ (Fig. 6(a)), while redox peaks become more evident after adding the Mn element. Moreover, the redox peak location varies with different Mn concentrations. The redox peaks in CV curves are mainly associated with the faradaic redox behaviour. The Co_3_O_4_@MnO_2_-3 electrode has a strong CV curve, exhibiting its maximum capacitance. It has been revealed that when the scan rate increases with current increases, the shape of the CV curves follows a similar pattern. The appearance of redox peaks and the deviation of the curves indicate that the storage mechanism is owing to the faradaic redox behaviours. The redox peaks are caused by electrolyte cations intercalating or de-intercalating in MnO_2_ nanosheets, which relates to eqn (4).^48^ The electrode material absorbs K^+^ ions from the electrolyte during charging. Then, K^+^ ions are released from the electrode material and released to the electrolyte during discharge. The cathodic peaks shifted towards lower negative potential due to polarization with increasing scan rates.

4 MnO_2_ + M^+^ + e ↔ MnOOM (M^+^ = K^+^)

**Fig. 7:**
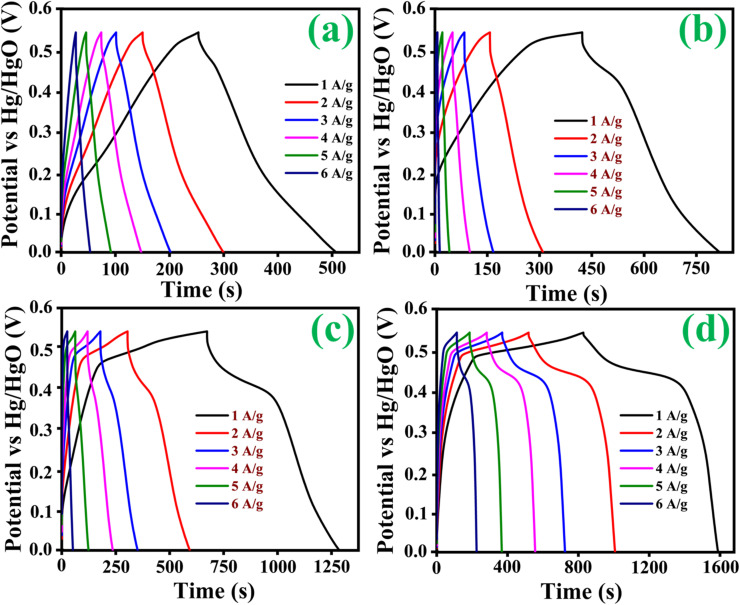
Chronopotentiometry curves of (a) Co_3_O_4_, (b) Co_3_O_4_@MnO_2_-1, (c) Co_3_O_4_@MnO_2_-2 and (d) Co_3_O_4_@MnO_2_-3.

**Fig. 8:**
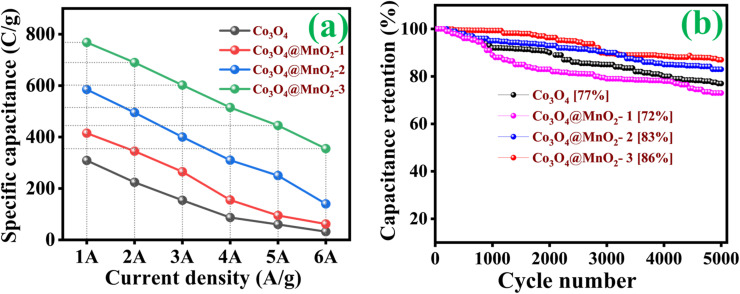
(a) Rate capacity and (b) cycle stability of various prepared electrodes.

CP curves demonstrated the typical faradaic behaviour of all prepared electrodes in the charge storage process at various current densities (1, 2, 3, 4, 5 and 6 A g^−1^). Discharge curves are nearly symmetric in pattern, with a slight *IR* drop at the beginnings of discharge, implying high redox reversibility. As clearly observed in the CP results shown in Fig. 7(a–d), eqn (1) follows. The Co_3_O_4_@MnO_2_-3 electrode reveals a longer discharge duration than the other prepared electrodes and archives specific capacitance around 768 C g^−1^ at 1 A g^−1^ current density shown in Fig. 7(d). This maximum specific capacitance is ascribed to the nearly complete redox reaction achieved by the Co_3_O_4_@MnO_2_-3 electrode material. Its initial *IR* drop is relatively low, confirming intense contact of the active material with the current collector. The other electrode materials are Co_3_O_4_, Co_3_O_4_@MnO_2_-1 and Co_3_O_4_@MnO_2_-2, which achieve lower capacitance around 309, 415 and 585 C g^−1^ at 1 A g^−1^ current density, respectively shown in Fig. 7(a–c). The specific capacitances of all electrode materials are measured and plotted in Fig. 8. (a) Using the CP curves, indicating that Co_3_O_4_@MnO_2_-3 (768 C g^−1^) is statistically superior to that of bare Co_3_O_4_ and other composite materials. The specific capacitance of Co_3_O_4_@MnO_2_-3 is significantly higher than the most often reported Co_3_O_4_-based electrode materials in Table 1.

**Table 1:** A comparison of Co_3_O_4_ composites-based electrode reports

S. no	Electrode material	Electrolyte	Specific capacitance (F g^−1^) or (C g^−1^)	Current density (A g^−1^)	Cycles	Capacity retention	Ref.
1	MOF-derived Fe_2_O_3_/MnO_2_	1 M KOH	908	1	8000	78%	49
2	Co_3_O_4_/carbon aerogel	2 M KOH	298.8	0.5	1000	82%	50
3	Hollow Co_3_O_4_@MnO_2_ cubic	1 M LiOH	413	0.5	2000	—	51
4	MOF derived porous Co_3_O_4_	2 M KOH	190	5	5000	71.42%	52
5	MOF-derived Co/C/Ni(OH)_2_	6 M KOH	952	0.5	10 000	84%	53
6	Ni-MOF on carbon cloth by ZIF-derived Co_3_O_4_	4 M KOH	1416	1	3000	90%	54
7	MOF-derived Co_3_O_4_/NiCo_2_O_4_	6 M KOH	770	1	10 000	70%	55
8	MOF-derived porous NiCo_2_O_4_ nanoparticle	1 M KOH	684	0.5	3000	86%	56
9	MOF-derived Co_3_O_4_–C/Ni_2_P_2_O_7_	3 M KOH	2537.78	2	3000	88.5%	57
10	NiO/Co_3_O_4_	6 M KOH	405	1	1000	97.4%	58
11	RGO/Co_3_O_4_	6 M KOH	546	0.5	10 000	90%	59
12	**Co**_**3**_ **O**_**4**_ **decorated with MnO**_**2**_ **nanosheets**	**1 M KOH**	**768 C g** ^**−1**^	**1**	**5000**	**86%**	**This work**

Fig. 8(b) shows that the cycling stability of all electrodes was examined for 5000 cycles at a constant current density of 6 A g^−1^. The Co_3_O_4_, Co_3_O_4_@MnO_2_-1, Co_3_O_4_@MnO_2_-2 and Co_3_O_4_@MnO_2_-3 exhibit cycling stability around 77, 72, 83 and 86%, respectively. The Co_3_O_4_@MnO_2_−3 electrode suggests high cycling stability and electrical conductivity compared to other electrodes. Furthermore, specific capacitance increases during the first few cycles due to the electrode material's activation influence and increased mobility of the surface charge and electrolyte ions.^60,61^ The relationship between peak current and sweep rate is determined to understand the charge storage kinetics process of all electrodes in 1 M KOH electrolyte. The peak current (*I*) measured from CV curves at various scan rates is calculated using the power-law equation.^61–63^

5 *i* = *av*^b^

**Fig. 9:**
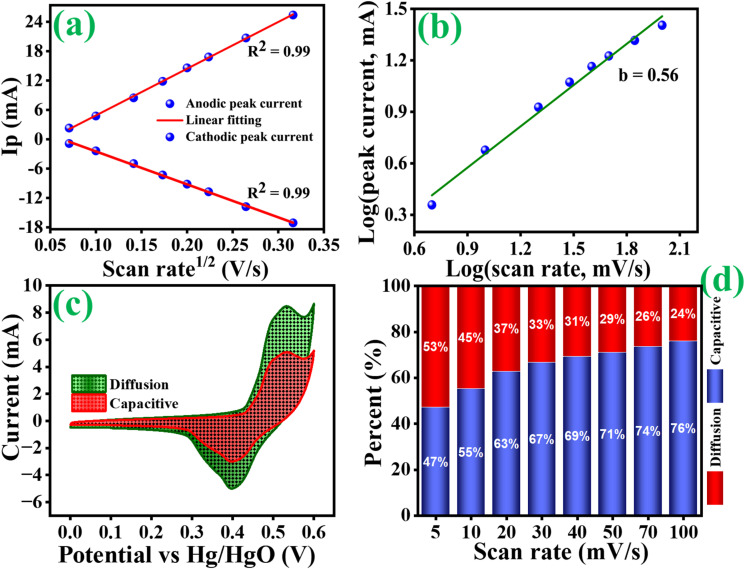
(a) Anodic and cathodic peaks as a function of scan rate, (b) slope for log of peak currents as a function of log of scan rates, (c) percentage of capacitance contribution to charge storage at 5 mV s^−1^ and (d) histogram of capacitance contribution ratio at different scan rates of Co_3_O_4_@MnO_2_-3 (d).

**Fig. 10:**
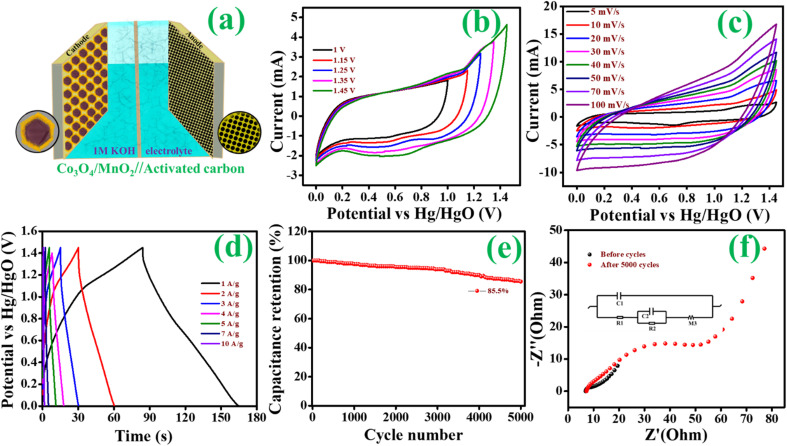
(a) Hybrid supercapacitor based on Co_3_O_4_@MnO_2_//AC electrode, (b) CV curves for the HSC at different potential windows, (c) CV curves at a different scan rate, (d) CP curves at different current densities, (e) the cycle stability and (f) EIS measurements of HSC.

6
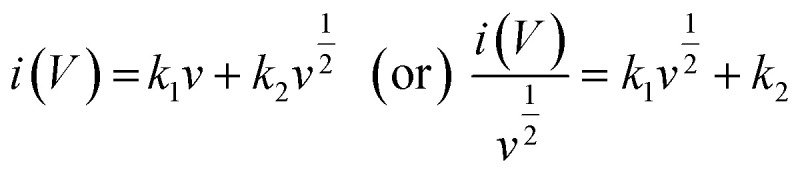
where *ν* is the scan rate, *i* is the peak current, *a*, *b*, *k*_1_ and *k*_2_ are adjustable parameters and *i*(*V*) is the current response at a fixed potential *V*. The square root of the scan rate and the corresponding current response correlates to the diffusion-controlled and capacitive control processes. The value *b* = 1 implies that the capacitive-controlled charge storage mechanism provides a rapid capability primarily responsible for the power density usually seen in carbon-based materials. In contrast, *b* = 0.5 suggests a diffusion-controlled charge storage mechanism. The anodic and cathodic peaks for the Co_3_O_4_@MnO_2_−3 are shown in Fig. 9(a). The linear relationship obtained illustrates the diffusion characteristics of the materials. The anodic and cathodic peak current values contain an *R*-square value close to one, indicating that the material has redox behaviour, which is one of the criteria for battery-type electrode material. The slope in Fig. 9(b) is 0.55, indicating the pure diffusion-controlled and battery-type electrode.^63^ Dunn's method can quantify the significant contribution of the diffusion and the capacitance mechanism. This approach allows for the quantitative determination of the CV curves contributed by the capacitive and diffusion control processes at varied scanning speeds. At a scan speed of 5 mV s^−1^, the red portion of the CV curve in Fig. 9(c) reflects the contribution ratio (47%) occupied by the capacitance control mechanism and the contribution ratio (53%) occupied by the diffusion control mechanism. In Fig. 9(d), the percentage of capacitance and diffusion contribution at each scan rate is given as a histogram. The capacitance contribution increases with increasing the scanning speed because capacitance control's surface effect is a quick process. The hybrid supercapacitor is represented in the diagram Fig. 10(a); the electrodes are Co_3_O_4_@MnO_2_ as cathode, activated carbon (AC) as an anode, and 1 M KOH as an electrolyte. According to prior research, the hybrid supercapacitor made of carbon-supported materials has a high energy and power density. Because of its high porosity and conductivity, activated carbon (AC) is used as a negative electrode. Its broad potential window and good specific capacitance allow absorbing more ions from the electrolyte.^64–67^ Furthermore, based on the above CV and CP results, Co_3_O_4_@MnO_2_-3 is assigned as a positive electrode for the two electrode systems. The operating potential window for the two electrode systems of Co_3_O_4_@MnO_2_-3//AC is performed by combining both electrodes. As shown in Fig. 10(a), the CV curves of Co_3_O_4_@MnO_2_-3, activated carbon, and hybrid supercapacitor electrodes were first recorded independently. The CV was performed at various potential windows for a hybrid supercapacitor to determine the ideal operating range of the potential window. As shown in Fig. 10(b), a broad potential window of 1.45 V was obtained. CV data was recorded at multiple scan rates ranging from 5 to 100 mV s^−1^ to examine the performance of the two-electrode system shown in Fig. 10(c). The CV curves are quasi-rectangular to a particular optimum value and then depart significantly at high potential. This variation from the typical rectangular form is caused by limiting ion transport on the electrode surface during redox processes at high scan rates. The CV curves remain unchanged even at higher scan rates, demonstrating that the hybrid supercapacitor has high-rate capabilities and stability. Additionally, no significant peaks have appeared, which indicates the hybrid supercapacitor exhibits dominating capacitive behaviour. Still, a more prominent knob in the curves indicates the existence of faradaic chemical processes. Charge discharge curves for the two-electrode system are also presented in Fig. 10(d), which shows the charge storage of the hybrid supercapacitor. The CP curves are neither triangular nor humped in the voltage window range of 0 to 1.45 V, but rather a combination of both types. At various current densities, the charge–discharge curves are essentially symmetric. The low *IR* drop appears to confirm the low internal resistance and good rate capability and validate the high cycle stability of the material. Stability studies are essential to provide insight into the material's lifetime. In this study, the hybrid supercapacitor is subjected to 5000 charge–discharge cycles at 6 A g^−1^ current density (Fig. 10(e)). The hybrid supercapacitor using Co_3_O_4_@MnO_2_ and activated carbon exhibited 85.5 percent capacity retention after 5000 charge–discharge cycles. The energy density of a hybrid supercapacitor is a significant indicator for determining its energy storage ability. The energy density and power density can be calculated from eqn (2) and (3). The Co_3_O_4_@MnO_2_//AC hybrid supercapacitors in the voltage window from 0 to 1.45 V provide a maximum energy density of about 60.17 W h kg^−1^ at a power density of around 2674.37 W kg^−1^. The prominent energy storage properties of Co_3_O_4_@MnO_2_//AC hybrid supercapacitor are mainly the vertically aligned nanosheets like Co_3_O_4_@MnO_2_ electrode provide good specific capacitance in a wide voltage window. The Nyquist plot of hybrid supercapacitors given in Fig. 10(f) identified the stability of hybrid supercapacitor before and after 5000 cycles. At low frequencies, the impedance rises substantially. It becomes nearly vertically parallel to the imaginary *y*-axis, indicating that the hybrid supercapacitor is pure capacitive. The charge transfer resistance (*R*_ct_) at the electrode–electrolyte interface is represented by the small semicircular part in the high-frequency region for after stability, which is combined with intrinsic resistance (*R*_s_) due to ionic resistance of the electrolyte and intrinsic resistance of the current collector. The equivalent circuit corresponding to the EIS data (inset) shows a slight increase of *R*_ct_ from 6.8 to 7.6 Ω obtained after 5000 cycles.

## 4. Conclusion

In conclusion, we effectively synthesized vertically aligned nanosheets like Co_3_O_4_@MnO_2_ with a distinct core–shell structure employing Co_3_O_4_ synthesized by sacrificing the ZIF-67 template as the precursor. As a result of the dense MnO_2_ nanosheets on the surface covering, the specific capacitance of Co_3_O_4_@MnO_2_-3 reveals around 768 C g^−1^, approximately two times that of the bare Co_3_O_4_, and exhibited good cycle stability and the porous structure of the material has a excellent BET surface area of 160.8 m^2^ g^−1^. Furthermore, a unique hybrid supercapacitor with positive and negative electrodes has been constructed with Co_3_O_4_@MnO_2_-3 and activated carbon, respectively. The hybrid supercapacitor provides high specific capacitance and long cycle life. Meanwhile, the energy and power densities were 60.17 W h kg^−1^ and 2674.37 W kg^−1^, respectively. This method provides a compelling alternative for preparing MOF-derived Co_3_O_4_-based composites as high-performance supercapacitor electrodes.

## Conflicts of interest

There are no conflicts to declare.

## Supplementary Material

RA-012-D2RA05603H-s001
