# Supplementary material for: Decorating MnO2 nanosheets on MOF-derived Co3O4 as a battery-type electrode for hybrid supercapacitors
Source: RSC Adv. 2022 Oct 11;12(44):28818–30. doi: 10.1039/d2ra05603h (PMC9552862; doi:10.1039/d2ra05603h)
Supplement: RA-012-D2RA05603H-s001 [file RA-012-D2RA05603H-s001.pdf]

Electronic Supplementary Material (ESI) for RSC Advances.
This journal is © The Royal Society of Chemistry 2022

Supplementary information:

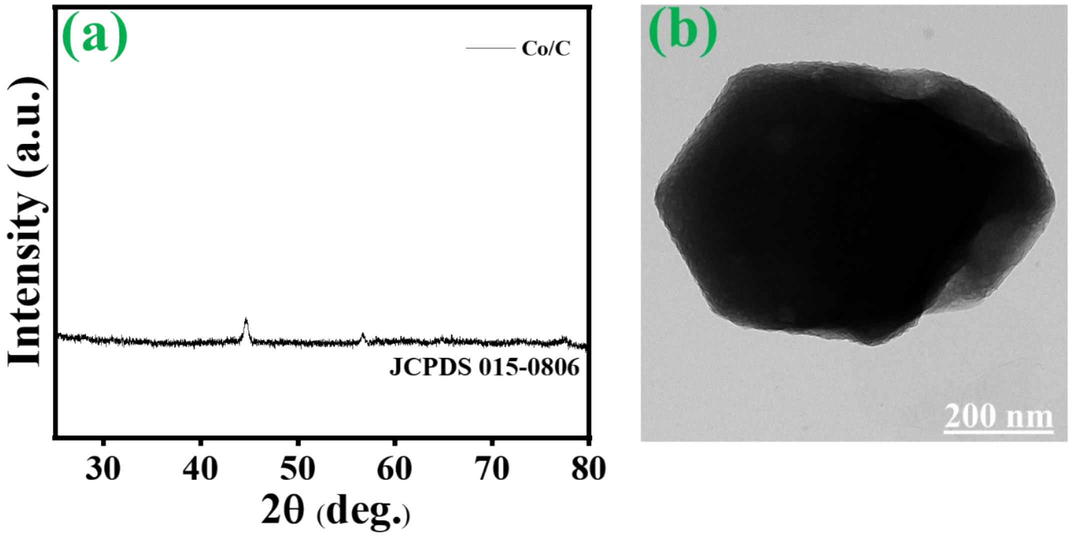

Fig. S1. (a) XRD pattern of intermediate Co/C. (b) TEM image obtained for Co/C.

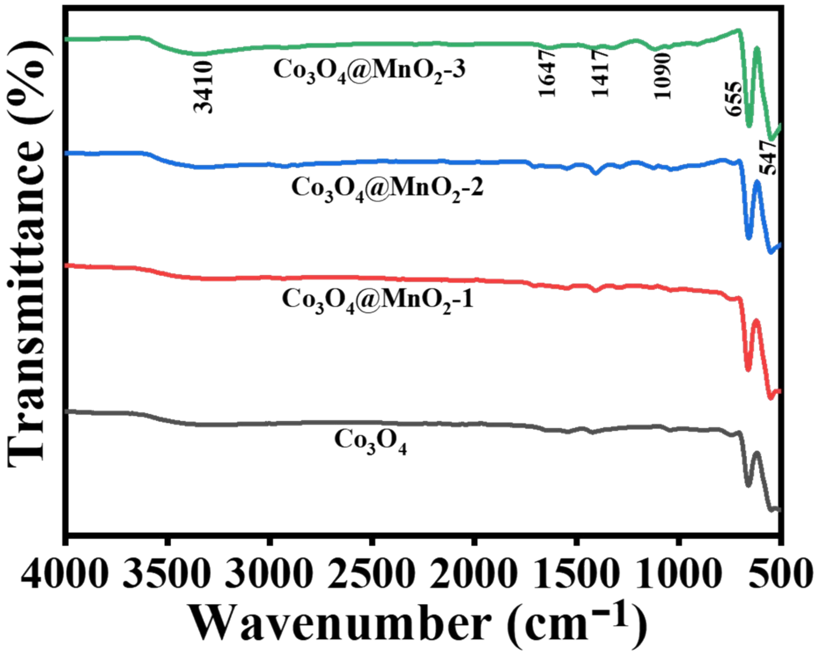

Fig. S2. FTIR analysis of Co_3O_4, Co_3O_4@MnO_2-1, Co_3O_4@MnO_2-2 and Co_3O_4@MnO_2-3

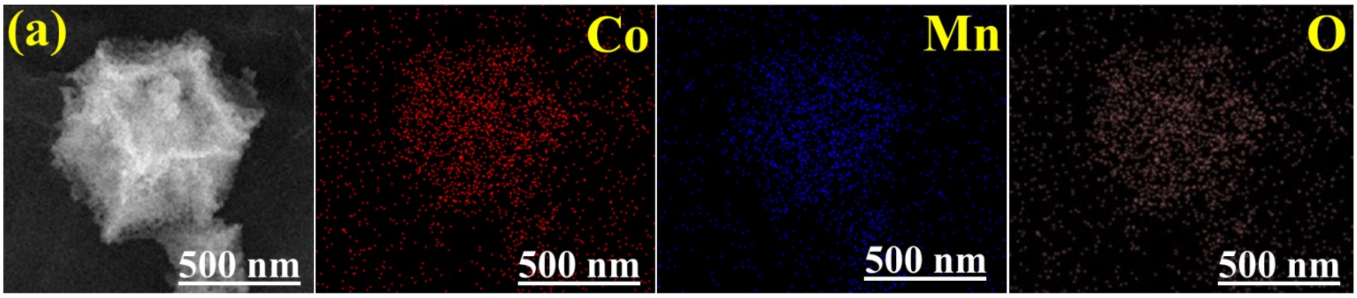

Fig. S3. Elemental mapping of Co_3O_4@MnO_2-3
